# Bradyarrhythmias: First Presentation of Arrhythmogenic Right Ventricular Cardiomyopathy?

**DOI:** 10.14740/jocmr2012w

**Published:** 2015-02-09

**Authors:** Danielle E Burghouwt, Janneke AE Kammeraad, Paul Knops, Frederik A du Plessis, Natasja MS de Groot

**Affiliations:** a Department of Cardiology, Erasmus Medical Center, Rotterdam, The Netherlands; b Department of Pediatric Cardiology, Erasmus Medical Center, Rotterdam, The Netherlands

**Keywords:** Arrhythmogenic right ventricular cardiomyopathy, Bradyarrhythmia, Atrioventricular conduction block, Pediatric patient

## Abstract

Arrhythmogenic right ventricular cardiomyopathy (ARVC) is a disorder characterized by progressive replacement of myocardial cells by fibro-fatty tissue giving rise to ventricular tachyarrhythmias. In this case report, we describe a pediatric patient with sinoatrial arrests and second degree atrioventricular conduction block several years before ARVC became apparent. These findings suggest that bradyarrhythmias can also be the first expression of ARVC.

## Introduction

Arrhythmogenic right ventricular cardiomyopathy (ARVC) is a disease characterized by progressive replacement of myocardial cells by fibro-fatty tissue [1-3]. The diagnosis is made with the Task Force Criteria [1]. Fibro-fatty replacement initially involves only the right ventricle, but may extent to the left ventricle as well [2, 3]. The disease is associated with ventricular tachyarrhythmias which can result in syncope or even sudden cardiac death [2-5]. However, we present a pediatric patient with bradyarrhythmias who is diagnosed with ARVC several years later.

## Case Report

**Figure 1:**
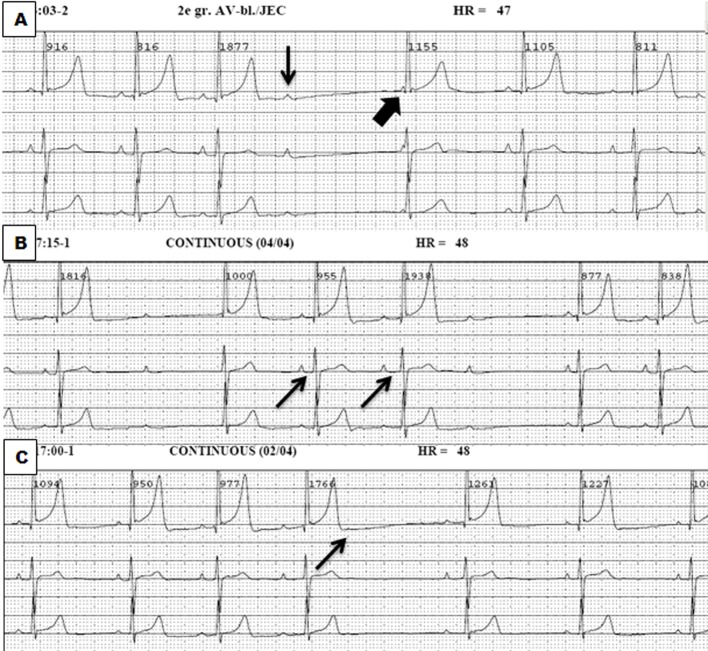
Holter recordings demonstrating a second degree atrioventricular block type II (thin arrow), sometimes followed by a junctional escape complex (thick arrow) (A), second degree atrioventricular block type I (B) and sinus arrests (C).

A 13-year-old male was referred to the pediatrician as atrioventricular conduction abnormalities were coincidentally observed during preoperative screening for dental surgery. His resting electrocardiogram (ECG) showed no other abnormalities. The 24-h Holter monitoring revealed frequent sinoatrial arrests (N = 77, up to 2.75 s) and a second degree atrioventricular conduction block type I and II (Fig. 1). No ventricular events were recorded. He did not have any complaints nor did he ever have a syncope. He participated in school gymnastics without any restraints. Physical examination was normal and echocardiographic examination also revealed no abnormalities. His family history was positive for cardiovascular disease. His grandmother had a myocardial infarction at the age of 75 years and his uncle at the age of 45 years. Another uncle died suddenly at the age of 30 years. Both uncles were brothers of his mother.

**Figure 2:**
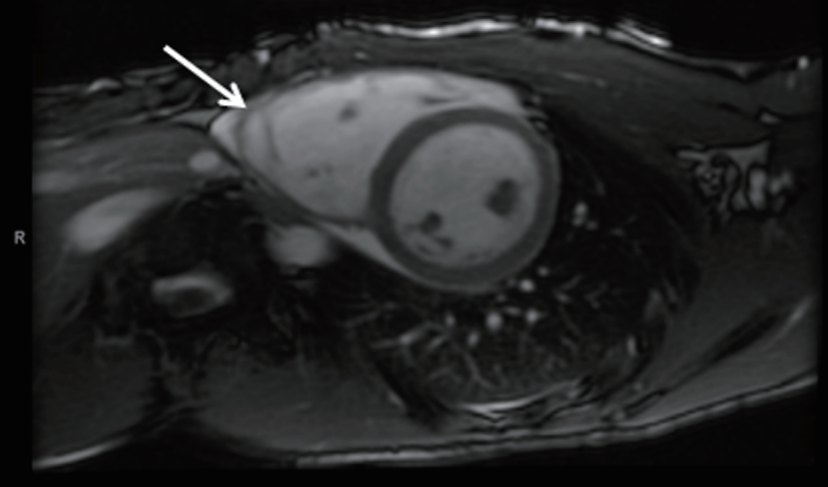
MRI in axial view shows the heart in diastole with a dilated right ventricle and trabeculation (arrow).

**Figure 3:**
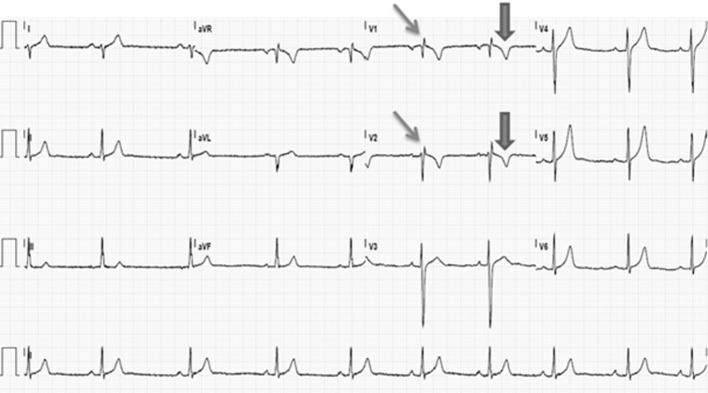
ECG recorded at the age of 17 years, demonstrating sinus arrhythmia, 54 bpm, right axis, PR-interval 164 ms, QRS-interval 93 ms, QTc-interval 348 ms. No epsilon waves. Negative T in V1 and V2 (thick arrows). Incomplete right bundle-branch block (thin arrows).

At the age of 17 years, 24-h Holter monitoring again showed a second degree atrioventricular conduction block type I and sinoatrial arrests (N = 224), but now with increased duration up to 3.48 s. The only ventricular events were four premature ventricular beats. During the following visit to the outpatient clinic, he complained of excessive exhaustion, dizziness and dyspnea during exercise and one episode of syncope. Additional cardiac evaluation included a signal averaged ECG and cardiac imaging. The signal averaged ECG was positive as two of the three parameters are abnormal including a low-amplitude signal duration of 44 ms (normal duration < 38 ms), and a root-mean-square voltage in the last 40 ms of the QRS of 12 µV (normal value: > 20 µV). The MRI showed a dilated, right ventricle (RVEDV/BSA: 133 mL/m^2^) with an ejection fraction of 41% and dyskinesia of the right ventricular anterior wall (Fig. 2). The ECG revealed inverted T waves in leads V1 and V2 in the presence of an incomplete right bundle-branch block (Fig. 3). Hence, the diagnosis of ARVC could now be made as one major and two minor criteria were fulfilled [1]. Because of his dysrhythmias, a DDD pacemaker was implanted, after which all his complaints resolved.

## Discussion

In this case report, we describe a pediatric patient with a second degree atrioventricular conduction block and sinoatrial arrests years before ARVC became overt. He presented with a coincidentally found second degree atrioventricular block type I at the age of 13 years and remained asymptomatic till the age of 17 years. Then he becomes symptomatic and a DDD pacemaker is implanted. Additional cardiac evaluation revealed that this patient at that moment fulfilled the criteria for ARVC.

ARVC is characterized by progressive replacement of myocardial cells by fibro-fatty tissue, initially only in the right ventricle, but it may extent to the left ventricle as well [2, 3]. The estimated prevalence is 1:5,000 individuals [4]. Genetic alterations underlying ARVC are often mutations in one of the cardiac desmosome genes [5]. These mutations are known for their phenotype diversity within families [6]. The golden standard for diagnosing ARVC is an endomyocardial biopsy demonstrating fibro-fatty replacement [1, 2]. ARVC is associated with ventricular tachyarrhythmias giving rise to syncope or sudden cardiac death. From the sudden cardiac deaths caused by ARVC, 10% of the patients were under the age of 18 years [3]. Premature ventricular beats or (non-) sustained ventricular tachycardias in these patients often originate from the right ventricle [2-5]. Our patient, however, presented with a second degree atrioventricular block type I and II, sinoatrial arrests and no ventricular tachycardias. There was no suspicion of ARVC until he became symptomatic.

Tabib et al examined 1,930 cases within the age range of 1 - 65 years out of 14,000 autopsies who had an “unsuspected sudden cardiac death (USCD)”, defined as no extra-cardiac abnormalities and no cardiac history [3]. After examination, 200 cases of the USCD were post-mortem diagnosed with ARVC. The His bundle and bundle branches were histologically examined and stained with hematein-phloxine-safran. In 68% of the ARVC patients, the conduction system was infiltrated with fibrotic (54.3%) or adipose tissue (8.1%) or a combination of both (5.6%). In a control group of 187 cases with a pulmonary cause of death, less than 5% of the cases had infiltration of fibrotic and/or adipose tissue in the conduction system. An important limitation of this study is that only the His bundle and bundle branches were examined. In line with our case report, further investigation towards the sinoatrial and atrioventricular node could be interesting. Tabib et al also reported on a patient who suddenly died because of a complete atrioventricular conduction block while recording an ECG and was post-mortem diagnosed with ARVC [3]. Based on this study, it can be suggested that the cardiac conduction system in our patient was also infiltrated by fibrotic/fatty tissue resulting in atrioventricular conduction abnormalities.

Peters et al investigated syncopes in patients diagnosed with ARVC. The prevalence in this population was 10-20% [7]. The minority of the ARVC patients had syncopes without documented ventricular tachyarrhythmias. There were also no ventricular tachycardias inducible during electrophysiological studies. This study demonstrated that an atrioventricular conduction block is the most important mechanism of syncope in ARVC patients after exclusion of documented or inducible monomorphic ventricular tachycardia [7]. From 37 patients with unexplained syncopes examination revealed either complete high-grade atrioventricular block (N = 3) or intermittent atrioventricular block type II and/or III (N = 4) [7]. It is generally assumed that (lethal) arrhythmias in ARVC patients are ventricular tachycardias arising from the right ventricle, but the cardiac conduction system may also be involved. Based on our observations and data from literature, we hypothesize that our patient presented with bradyarrhythmias as a first expression of ARVC due to damage of the cardiac conduction system by fibrotic and/or fatty tissue. Absence of structural abnormalities during echocardiographic examination at the age of 14 years suggests that ventricular myocardium may not have been affected (yet), or only minimally. Hence, bradyarrhythmias can also be a (first) expression of ARVC.

This assumption raises the questions on how many ARVC patients died suddenly due to atrioventricular conduction abnormalities instead of ventricular tachyarrhythmias, and how many patients with conduction abnormalities actually may develop or already have ARVC. So far, sinoatrial involvement has rarely been described in ARVC. Morimoto et al observed fatty tissue in the sinoatrial node during autopsy in two ARVC patients [8]. Also, atrioventricular conduction abnormalities have only rarely been described in patients already diagnosed with ARVC. However, these rare descriptions show that atrioventricular conduction abnormalities may exist in ARVC patients [7, 9]. A pediatric patient presenting with bradyarrhythmias as a first expression of ARVC has to our knowledge never been described.

In conclusion, we described a 13-year-old male who presented with a coincidentally found asymptomatic second degree atrioventricular conduction block and sinoatrial arrests. At the age of 17 years, he became symptomatic and further cardiac evaluation revealed ARVC.
